# Different spatial pattern of municipal prostate cancer mortality in younger men in Spain

**DOI:** 10.1371/journal.pone.0210980

**Published:** 2019-01-25

**Authors:** Lara Rodriguez-Sanchez, Pablo Fernández-Navarro, Gonzalo López-Abente, Olivier Nuñez, Nerea Fernández de Larrea-Baz, Jose Juan Jimenez-Moleón, Álvaro Páez Borda, Marina Pollán, Beatriz Perez-Gomez

**Affiliations:** 1 Urology Department, Fuenlabrada General Hospital, Fuenlabrada, Spain; 2 Rey Juan Carlos University, Móstoles, Spain; 3 Cancer & Environmental Epidemiology Unit, Department of Epidemiology of Chronic Diseases, National Centre for Epidemiology, Carlos III Institute of Health, Madrid, Spain; 4 Consortium for Biomedical Research in Epidemiology & Public Health (CIBER en Epidemiología y Salud Pública—CIBERESP), Madrid, Spain; 5 Instituto de Investigación Biosanitaria de Granada (ibs.GRANADA), Granada, Spain; 6 Complejo Hospitales Universitarios, Granada, Spain; 7 Department of Preventive Medicine and Public Health, University of Granada, Granada, Spain; 8 Cardiovascular & Metabolic Diseases Unit, Department of Epidemiology of Chronic Diseases, National Centre for Epidemiology, Carlos III Institute of Health, Madrid, Spain; Universidade Nova de Lisboa Instituto de Higiene e Medicina Tropical, PORTUGAL

## Abstract

**Background:**

Prostate cancer (PC) primarily affects elderly men. However, the specific features of cases diagnosed at younger ages (<65 years) suggest that they may represent a different clinical subtype. Our aim was to assess this suggestion by contrasting the geographical PC mortality and hospital admissions patterns in Spain for all ages to those in younger men.

**Methods:**

The Spanish National Institute of Statistics supplied data on PC mortality, hospital admission, and population data. We estimated the expected town-specific number of deaths and calculated the standardized mortality ratios. Spatial autoregressive models of Besag-York-Mollié provided smoother municipal estimators of PC mortality risk (all ages; <65 years). We computed the provincial age-standardized rate ratios of PC hospital admissions (all men; <60 years) using Spanish rates as the reference.

**Results:**

A total of 29,566 PC deaths (6% among those <65 years) were registered between 2010–2014, with three high-mortality risk zones: Northwest Spain; Southwest Andalusia & Granada; and a broad band extending from the Pyrenees Mountains to the north of Valencia. In younger men, the spatial patterns shared the high risk of mortality in the Northwest but not the central band. The PC hospital discharge rates confirmed a North-South gradient but also low mortality/high admission rates in Madrid and Barcelona and the opposite in Southwest Andalusia.

**Conclusion:**

The consistent high PC mortality/morbidity risk in the Northwest of Spain indicates an area with a real excess of risk. The different spatial pattern in younger men suggests that some factors associated with geographical risk might have differential effects by age. Finally, the regional divergences in mortality and morbidity hint at clinical variability as a source of inequity within Spain.

## Introduction

Worldwide, prostate cancer (PC) is the second most common tumor among men, with an estimated 1.1 million new cases in 2012[1]. The steep increase in incidence in most countries in recent decades[2] is generally attributed to the increased detection of indolent prostate neoplasms (i.e. use of prostate-specific antigen [PSA] tests as screening tools)[3] a potentially increased exposure to unknown risk factors[4] accompanied by a sustained decrease in mortality[5], which, in Spain started in the late 1990s[6]. With an estimated 307,000 deaths in 2012, PC ranks fifth as the cause of cancer death worldwide among men and third among European and Spanish men[1,7]. Based on these figures, it is surprising that scarce evidence is available regarding PC etiology. To date, none of the main well-established risk factors -age, genetic susceptibility[8], black race, and familial history- are modifiable[4]. The association of this tumor with other factors such as chemical contaminants[9], diet[10], and medications[11], among others, is not yet clear.

PC is generally considered a disease of older men. In Spain, the mean patient age at death in 2015 was 80.6 years[12]. However, an increasing number of new diagnoses occur in men aged ≤65 years and the incidence in these age groups is increasing at a higher rate than those in the older age groups[2]. Some authors have suggested that PC affecting younger men may constitute a different clinical subtype; in this sense, genetic factors play a more relevant role in this subgroup[13], and several clinical studies have reported that early-onset advanced-stage PC has a poorer prognosis and lower survival than those of tumors diagnosed in older age groups[2,8,14]. As it has also been reported that the effect of some risk factors (i.e. selenium levels) might be higher among those with increased genetic predisposition[15], the relationship of PC in younger men with external exposures could differ from that of all cases.

Spatial studies and cancer atlases can be useful tools to suggest new etiological hypotheses for PC based on the geographical variability in the risk of death due to this cause[16], either related to the differential distribution of possible risk factors or to the variability in diagnostic and therapeutic strategies among areas. We utilized this strategy to compare the spatial distributions of municipal PC mortality in Spain in all men to the pattern observed for PC mortality in younger men to identify clues to provide data about early-onset PC. A different mortality spatial pattern in men under 65 years of age (those probably diagnosed with advanced tumors a decade earlier[17]) could suggest that some factors associated with the geographical risk might have differential effects by age group. Finally, we contrasted both patterns with the geographical distribution of PC-related hospital admissions in the same period to gain insight into the role that variability in medical practice may play in them.

## Material and methods

**Fig 1:**
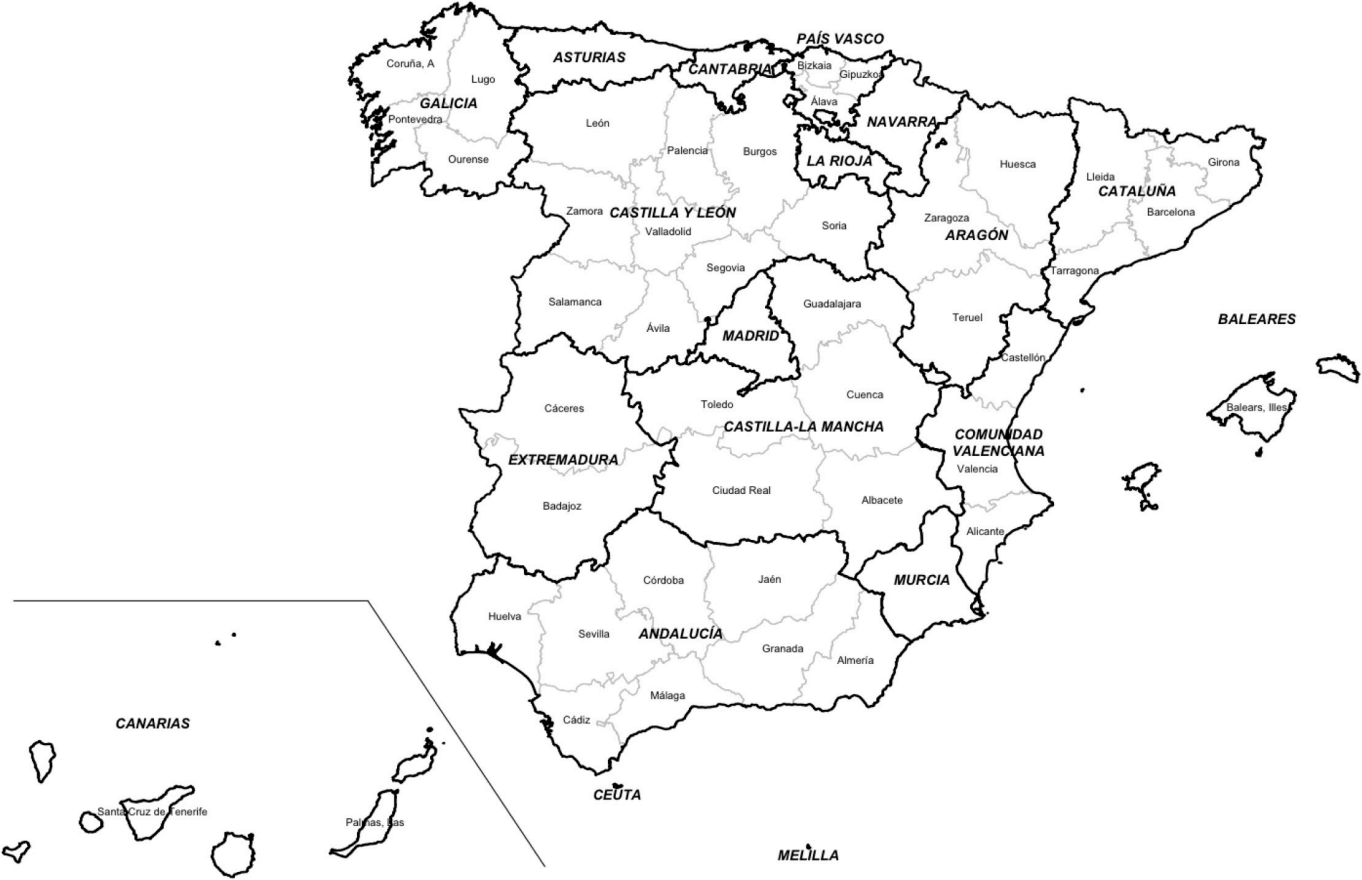
Regional and provincial administrative distributions within Spain.

Fig 1 shows the Spanish regional and provincial administrative distributions to help the reader to better understand the description of the results.

### Prostate cancer mortality

We mapped the municipal relative risks (RR) of PC mortality (smoothed standardized mortality ratios) in Spain for the period 2010–2014 and the distributions of posterior probabilities (PP) of having an RR>1 for a) all ages and b) those younger than 65 years of age. The RRs were calculated using spatial models including observed and expected cases of PC death at the municipal level. We also applied this methodology to men ≥65 years; the resulting map is presented as Supporting Information (S1 Fig) because of its redundancy with the map for all ages.

#### Observed and expected cases

The National Statistics Institute (Instituto Nacional de Estadística-INE) provided data on a) individual death entries between 2010 and 2014 in men in Spain corresponding to PC (International Classification of Diseases, 10th Revision: C61), broken down by municipality (n = 8,097), and b) the Spanish municipal roll male population data by town and age (18 age groups) in 2012, the midpoint of the study period. We calculated the numbers of expected cases by multiplying the overall Spanish age-specific mortality rates for the five-year study period by each town’s person-years (2012 population*5). Afterward, standardized mortality ratios (SMRs) were computed as the ratios of the observed to the expected deaths.

#### Municipal relative risks (RRs)

Municipal smoothed SMRs (RRs) were calculated using the conditional autoregressive model proposed by Besag, York and Mollié[18], based on fitting a Poisson spatial model with observed cases as the dependent variable, expected cases as an offset, and two types of random effect terms: a) municipal contiguity (spatial term h_i_); and b) municipal heterogeneity (b_i_). The model takes the following form:

$$O_{i}∼Poisson(E_{i}\lambda_{i})$$

$$log(\lambda_{i})=\alpha +h_{i}+b_{i}$$

i=1:8097municipalities

Where λ_i_ is the RR in area i; O_i_ is the number of deaths in area i; α is the intercept quantifying the average mortality rate in all municipalities; and E_i_ is the expected number of cases. The models were fitted with “Integrated nested Laplace approximations” [19] (INLAs) as the tool for Bayesian inference, using the R-INLA package with the option of a simplified Laplace estimation of the parameters and the default specification for the distribution of the hyper-parameters in all the models. The spatial term (h_i_) is modelled using a conditional autoregressive structure and the heterogeneity term (b_i_), which corresponds to the unstructured residual, is modelled using an exchangeable prior. The criterion of contiguity was the adjacency of the municipal boundaries according to official INE maps.

### Prostate cancer morbidity

For the study of the geographical distribution of prostate cancer patient admissions, we decided to complement the 5-year period depicted in the mortality data (2010–2014) with the previous one (2005–2009) and to move the age limit to 60. We wanted to look at the same cohort of men in mortality and morbidity data; as relative survival in Spain for PC is close to 85% at 5 years[20], any man dead from PC would usually be treated in the hospital at least five or ten years before (i.e. 5–10 years younger). We used the Hospital Morbidity Survey[21] carried out by the INE to determine the number of hospital admission records in men having PC as main diagnosis at discharge (ICD9-CM: 185; ICD10: C61), as well as the corresponding population figures for the periods 2005–2009 and 2010–2014, broken down by province (n = 50, excluding Ceuta and Melilla cities due to their small populations) and age (18 age groups). We applied the corresponding elevation factors and calculated age-adjusted discharge rates per province for all patients as well as for those under 60 years at admission in each quinquennium; afterward, we mapped the admission rate ratios per province for the same strata, using the Spanish rates as the reference. The map corresponding to men > = 60 years is included as Supporting Information (S2 Fig).

R software was used to create maps and to perform all the previously mentioned statistical analyses.

## Results

**Fig 2:**
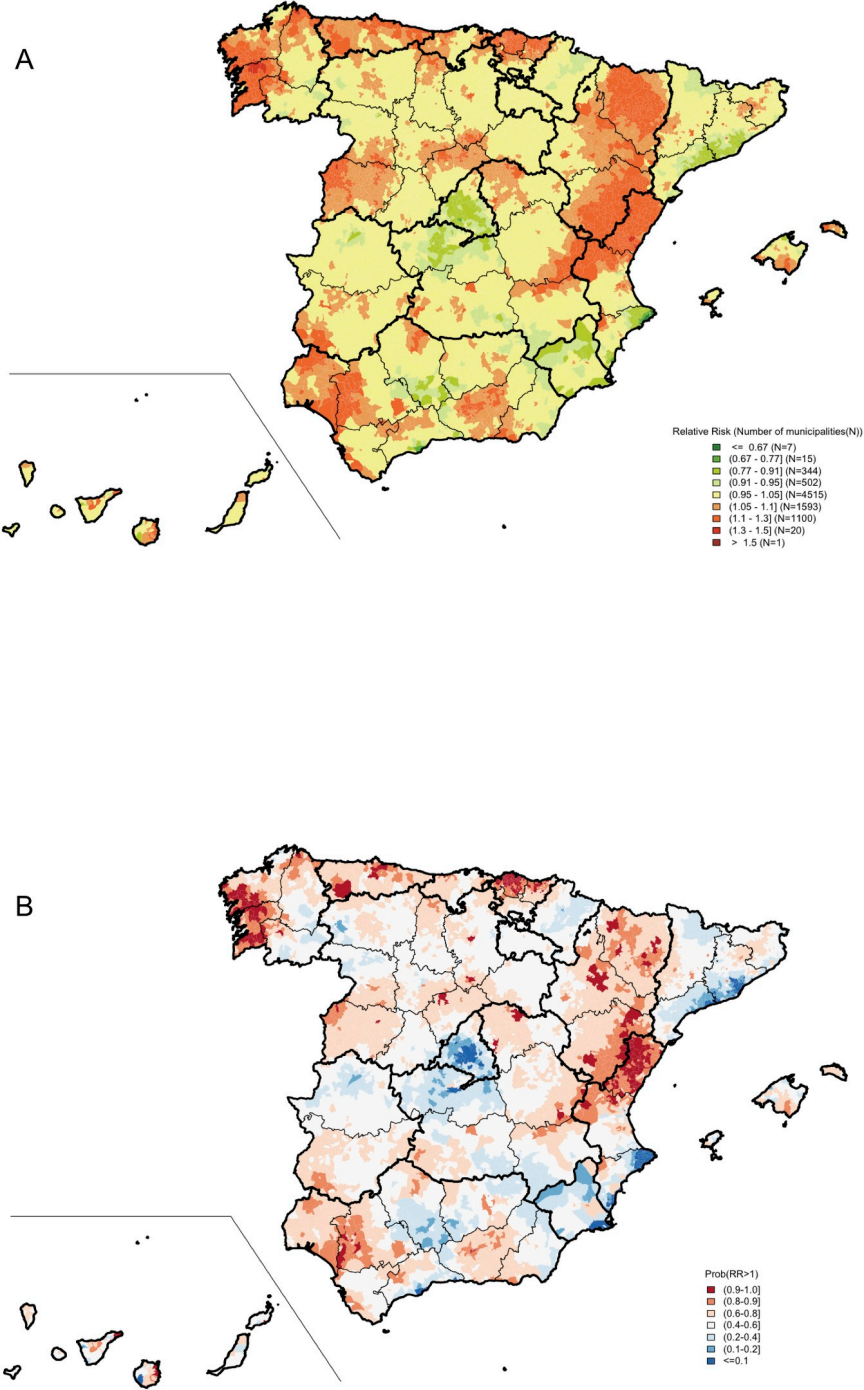
Prostate cancer mortality in Spain between 2010 and 2014 (all men). Municipal distributions of the relative risks of death (A) and posterior probabilities of having a relative risk greater than 1 (B).

**Fig 3:**
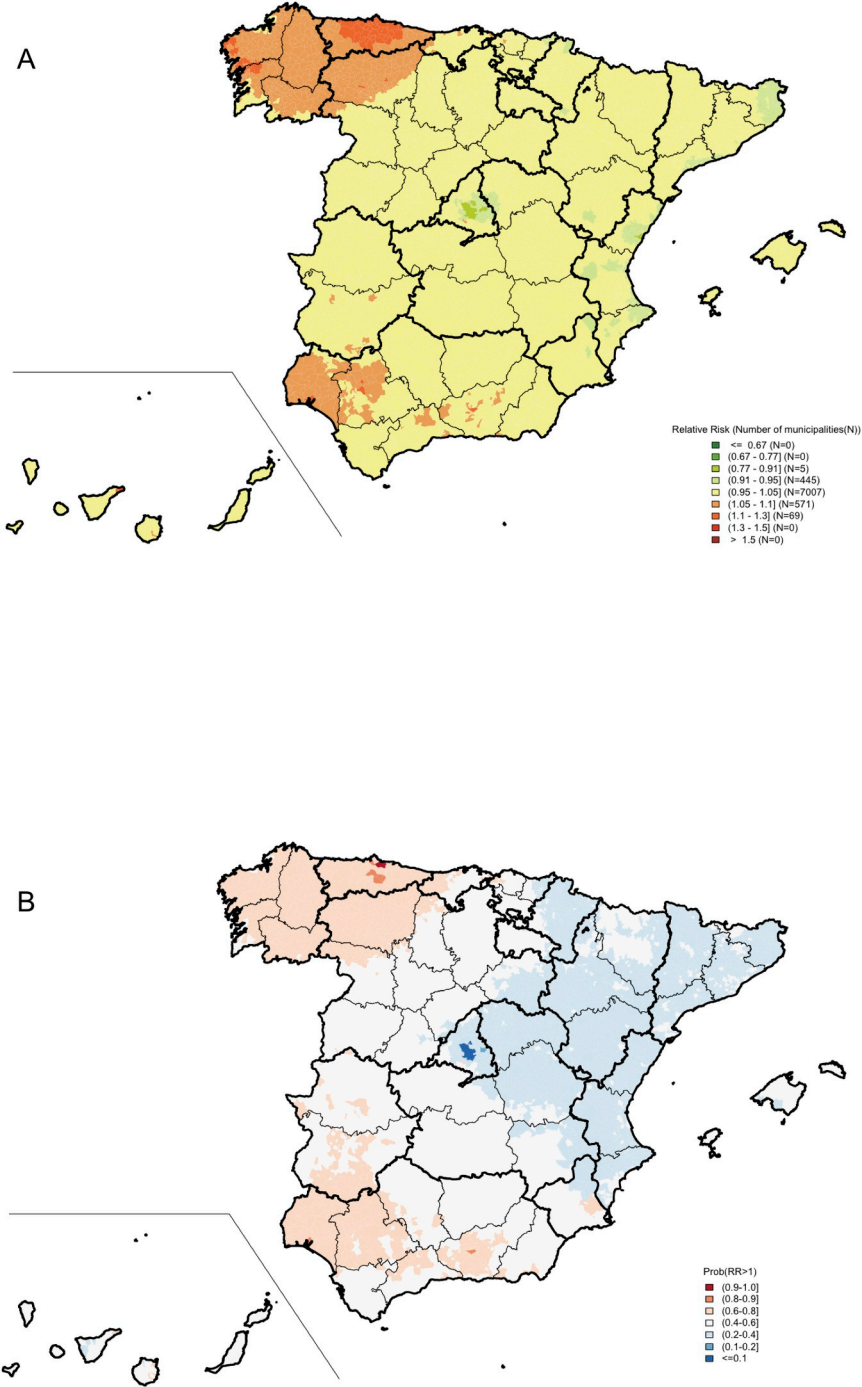
Prostate cancer mortality in Spain between 2010 and 2014 (men <65 years). Municipal distributions of the relative risks of death (A) and posterior probabilities of having a relative risk greater than 1 (B).

A total of 29,566 deaths due to PC (6% in men younger than 65 years old) were reported in 2010–2014. The mortality patterns are shown in Fig 2, which presents the spatial distributions of the risk of death due to PC for all PC cases, while Fig 3 shows this information for PC deaths in men <65 years of age. Each figure includes two maps: one depicting the municipal risk of death due to PC compared to the average in Spain and another representing the probability for each town of having an excess of risk (RR>1).

For all ages, municipal RR ranked from 0.46 to 1.54, and three areas could be considered high-mortality risk zones: a) northwest Spain, that is, the Atlantic and Cantabrian coasts, mainly Galicia, Asturias, and the Basque Country; b) southwest of Andalusia and Granada; and c) a long band crossing from the Pyrenees Mountains, through Aragon, to Castellon and the north of Valencia on the Mediterranean coast. In contrast, lower risks than the average of Spain were observed in Madrid, in the coastal areas of Tarragona, Barcelona, Alicante, and Murcia (Fig 2).

The geographical pattern in younger men (Fig 3) was less pronounced than the one observed for all men, with RR ranging from 0.88 to 1.24. In this case we also observed higher risk areas in the northwest, mainly in Galicia and Asturias, and southwest Spain (Huelva), but there was a striking difference with the pattern seen in Fig 2, including the absence of risk in Aragon, Castellon, Valencia, and in the Basque Country.

**Fig 4:**
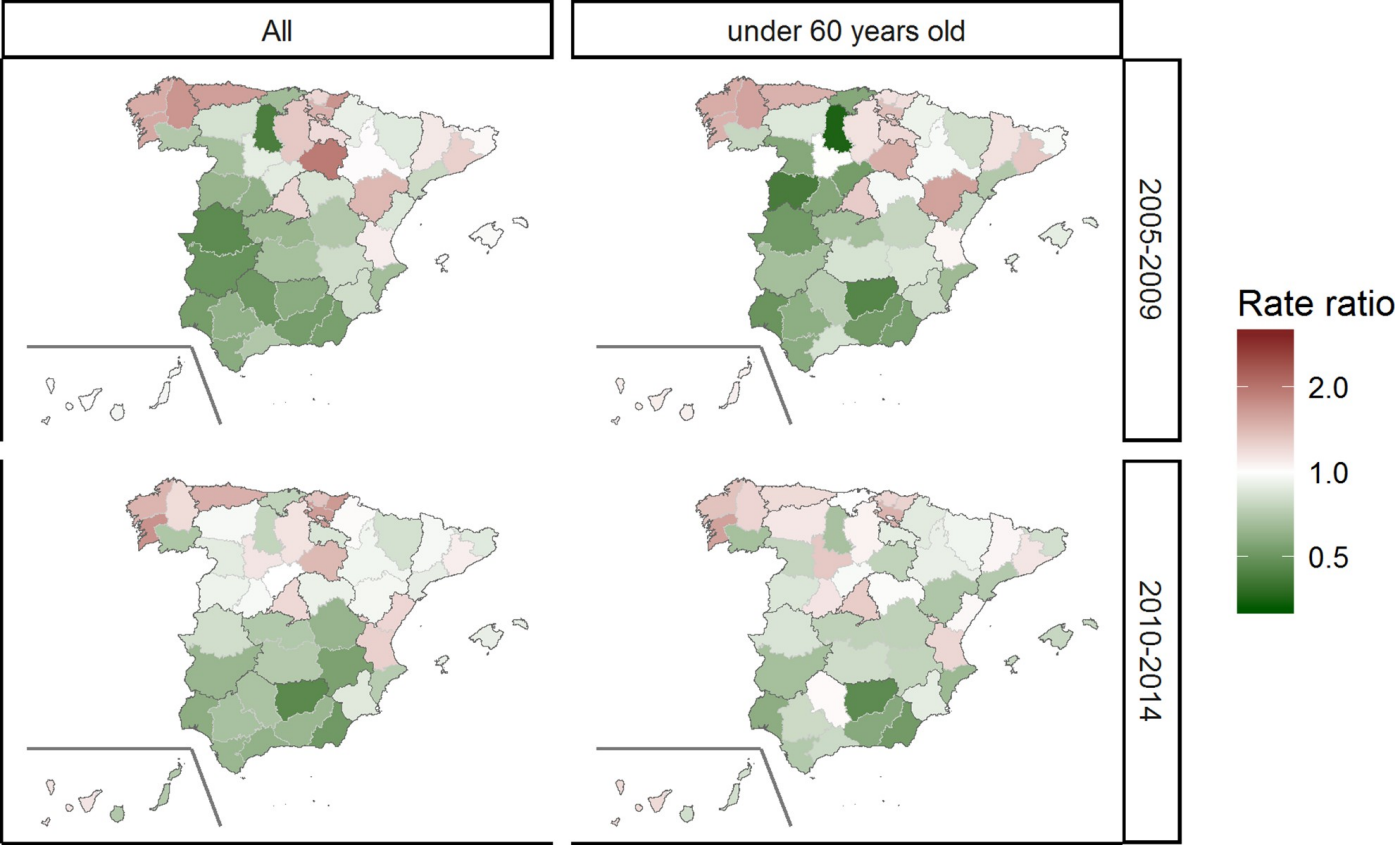
Provincial rate ratios of age-standardized hospital admissions in Spain between 2005 and 2014 with prostate cancer as the main diagnosis by 5-year period for all men and those <60 years of age.

The risks of hospital admissions due to PC are depicted in Fig 4. For the period 2005–2009 rate ratios varied between 0.43–1.92 in all cases and between 0.36–1.57 in men <60 years, while for 2010 to 2014 period, they ranged between 0.46–1.70 for all men and between 0.47–1.58 in younger men. Both maps presented a similar pattern, and showed an even clearer general north/south gradient than that observed for mortality rates. Thus, hospitals in the northwest provinces (Galicia, Asturias, and the Basque Country) had higher PC-related age-adjusted admissions rates than the average rate in Spain in both young and older patients; however, Extremadura, Andalusia (including the southwestern portion), and Castile-La Mancha had low rates of hospital admissions. Furthermore, there were slight excesses of hospital discharges in Madrid and Barcelona, which are areas with low mortality risks.

## Discussion

The spatial distribution of PC mortality by municipality in the period 2010–2014 in Spain showed high-risk zones in the north and the west, in line with period 2004–2008 [22], although with a more marked excess of mortality in the southwest of Andalusia. However, the most noteworthy result from the present study is the contrasting mortality patterns observed between all ages and those of younger men (<65 years) in the eastern part of the country.

Based on the survival figures for PC in Spain[22], our map of mortality due to PC before 65 years of age likely shows the spatial distribution of the risk of dying in men diagnosed with advanced tumors a decade earlier. PC tumors in men under 55 years of age are more likely to have been detected through PSA screening and to be of low grade; consequently, these patients have equivalent or even higher survival rates than the rates in older ones[23]. However, the subgroup of younger men with high-grade or metastatic tumors has a higher cause-specific mortality rate than those among men diagnosed at an older age[14,23]; this finding suggests that these tumors might constitute a different clinical entity[8,13] with specific biological features[24,25]. Our study supports the hypothesis that PC in younger men may have its own specificities, at least from an epidemiological point of view, as it showed a clear difference among spatial mortality patterns depending on age: for all cases, there was an excess risk in the eastern band from the Pyrenees to the north of the Valencian Community (Castellon), as well as in the Basque Country, which was absent in those who died before 65 years of age. These diverse spatial mortality patterns might derive from differences in a) incidence (variability in genetic predisposition, diagnostic strategies, or exposure to environmental causes); b) lethality (regional differences in the availability or quality of medical treatment or in the exposure to promoter risk factors), and c) death certificate quality.

Regarding genetic factors, genome-wide association studies (GWAS) have identified over 100 single nucleotide polymorphisms associated with the development of PC[26] and a family history of PC doubles the risk of disease development in first-degree relatives[27]; these factors appear to be associated with an increased risk among men under 65 years of age[28]. Unfortunately, there is no information about the geographical variability of genetic PC susceptibility; however, in a preliminary analysis of a PC polygenic risk score in the Spanish multi-province MCC-Spain PC case-control study, we found quite small differences among regions, suggesting that this factor may not be very relevant in the geographical diversity of risk.

A possible contributor to the spatial discrepancy by age, more related to variability in clinical practice, may be the effect of PC screening strategies on mortality statistics. In Spain, PC is among the well-certified tumors in death registries[29]; however, some authors have reported that the diagnosis of prostate cancer due to PC screening can lead practitioners to report more deaths as due to this cause (attribution bias)[30], an effect that is stronger in older men[31]. Thus, those areas more prone to PSA use would have a higher risk of PC-attributed death in all men, which would be less apparent when only younger men are considered. Unfortunately, we have no information regarding the date of introduction of PSA testing in Spain, or on its spread or prevalence across the country, which could help to test its effect on these geographical patterns[32].

PC is also a tumor with higher variability in its indication and types of treatment[33]. This is due to a combination of different social, organizational, and clinical factors, which may strongly influence the spatial distribution of PC risk. Even though Spaniards have universal public health coverage, the available data indicate that the rate of PC surgical interventions is higher in areas with better socioeconomic indicators[34]. The mortality risks in younger men present a higher homogeneity. This is probably associated with the low number of deaths in the younger group, but another plausible contributing explanation is a lower variability in the treatment of advanced cases in young patients, which usually implies a more aggressive clinical approach than that in elderly men[13] due to their *a priori* higher life expectancy.

An alternative hypothesis to explain the different spatial patterns by age could be the presence of environmental agents in these areas for which a sustained, chronic exposure could induce PC and which would be more likely to occur at advanced ages. Among the candidate environmental pollutants is arsenic, classified by the International Agency for Research on Cancer (IARC) as a possible carcinogen for the prostate[35]. In a recent study carried out by our group, we observed a significant association between topsoil levels of this carcinogen and total PC mortality in 1999–2008[36]. The arsenic soil level was higher in the northwest and southwest regions of Spain, as well as in the area of Castellon and south of Aragon. Also present in soil is selenium; its levels modulate selenium concentration in food, the main source of exposure in humans. According to observational studies, selenium is a putative protective factor for PC[37], although this association was not corroborated by the SELECT clinical trial[38]. In Spain, the topsoil concentrations of selenium are highly variable, but the highest levels are found in the northwest regions, including Galicia, the Valencian Community and, less noticeably, in the southwestern parts of the country[39], all areas with high PC mortality. Our data do not seem to support the preventive role of selenium exposure, at least at an ecological level.

Pesticides, which have also been related to PC occurrence[40,41], could also be involved in this pattern. In recent years (1999–2014), the use of pesticides in Aragon has increased by 22.6%[42]. Valencian Community and Andalusia are also among the regions with the highest use of pesticides, comprising around 13% and 30%, respectively, of the country's pesticide consumption[42]. Therefore, further research in this regard could be of great interest.

Among other possible factors that might help us to understand the distribution of PC risk, diet has always been a candidate, as Spain has a marked dietary diversity among regions. The World Cancer Research Fund (WCRF) report suggests that dairy products and calcium-rich diets might increase the risk of PC[43]. Milk consumption in 2008 was higher in the northwestern parts of the country (Asturias, Cantabria, and Basque Country) as well as in Extremadura and Castile-Leon, and very low in Andalusia, Catalonia, and Murcia[44]. In contrast, Galicia, Cantabria, Basque Country, and Castile-Leon are among the regions with the highest consumption of fresh fish[44], which has been associated with lower PC mortality[45]. The impact of diet on PC mortality may be more accurately studied if global dietary patterns are considered[10].

The North-West of Spain (Galicia and Asturias) deserve a specific comment. The excess of mortality in this part of the country among young men and all men, together with the consistently higher rates of hospital admissions in these areas, point to a real excess of PC risk. Obesity may play a role in this finding: both regions lead the adult overweight rate rankings[46] and, according to the WCRF[43], body fatness is likely to be associated with advanced-stage prostate tumors[47], although the mechanisms remain unclear. However, weight excess is also very high in Extremadura and Castile-La Mancha, where the risk of death due to PC was not especially striking[48].

The two provinces that include the main cities of Spain, Madrid and Barcelona, also merit attention: these regions presented very low mortality risks in both mortality maps, in contrast to their higher than average rates of hospital admissions; the contrary was observed in Huelva (west Andalusia), with high mortality and low admission rates. These discrepancies require additional study in more detail to evaluate the differences in the healthcare patterns among regions.

One of the strengths of this work is the novel approach to assess the distribution of PC mortality by separately evaluating the risks in the younger cases as well as the combined use of spatial mortality and morbidity that provides a more complete picture of PC risk in Spain. However, this report also has limitations inherent to all ecological studies. This kind of approaches are useful to develop new hypothesis, but the lack of age-specific geographical distributions of the possible risk factors (i.e. diet or arsenic exposure) precludes the possibility of testing for specific associations. Also, as we have mentioned, there might be variability in death certificate coding among areas; however, the coordinator role of the National Institute of Statistics and the quality control process at regional level probably attenuate the effect of this a source of error. Finally, the low number of deaths in the case of prostate cancer under 65 year old might lead to potentially oversmoothed risk maps. For this reason, the models have been parameterized according to the recommended specifications to minimize bias[19]

## Conclusions

In conclusion, the consistently high PC mortality and morbidity risk in the northwest of Spain point to a higher risk of PC in this region. The different spatial pattern in men under 65 years of age suggests that some factors associated with the geographical risk might have differential effects by age group. Finally, the divergences in mortality and morbidity patterns in some regions, such as Madrid, Barcelona, or Huelva, indicate that variability in the patterns of care could be a relevant source of inequity among Spanish regions.

## Supporting information

S1 Fig Prostate cancer mortality in Spain between 2010 & 2014 (Men > = 65 years old). Municipal distribution of relative risk of death (A) and municipal distribution of posterior probabilities of having a relative risk greater than 1 (B)
(TIFF)

S2 Fig Age-standardized hospital admissions due to prostate cancer in Spain (> = 60 years). Provincial rate ratio by 5-year period between 2005 & 2014.
(TIFF)

## Abbreviations

PC: prostate cancer
RR: municipal relative risks
PSA: Prostate Specific Antigen
INE: Instituto Nacional de Estadística
SMRs: standardised mortality ratios
ICD: international classification of diseases
INLA: Integrated nested Laplace approximations
IARC: International Agency for Research on Cancer
WCRF: World Cancer Research Fund
