# Supplementary figures and images for: Different spatial pattern of municipal prostate cancer mortality in younger men in Spain
Source: PLoS One. 2019 Jan 25;14(1):e0210980. doi: 10.1371/journal.pone.0210980 (PMC6347247; doi:10.1371/journal.pone.0210980)

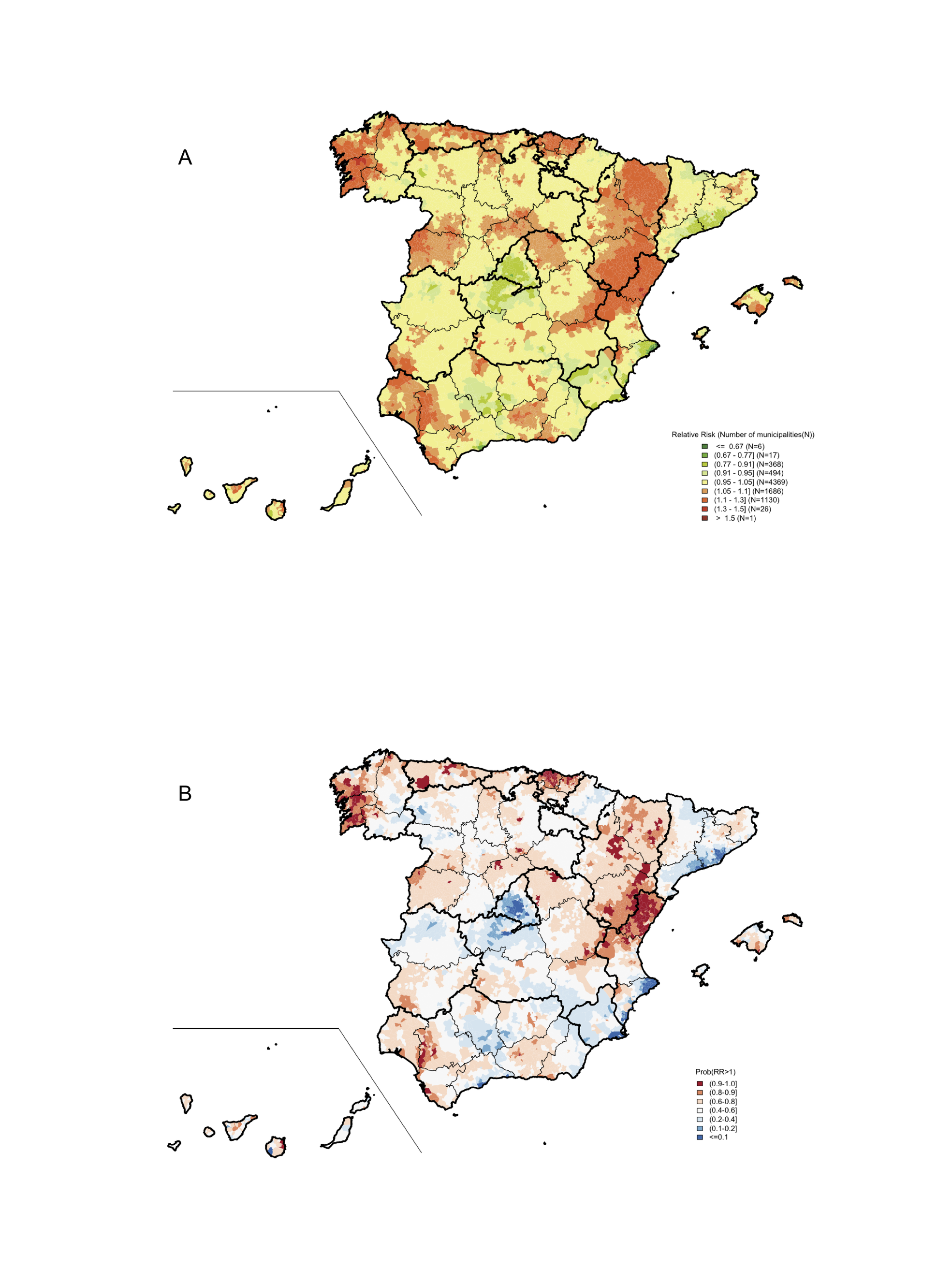

Supplement: S1 Fig — Municipal distribution of relative risk of death (A) and municipal distribution of posterior probabilities of having a relative risk greater than 1 (B) (TIFF) [file pone.0210980.s001.tiff]

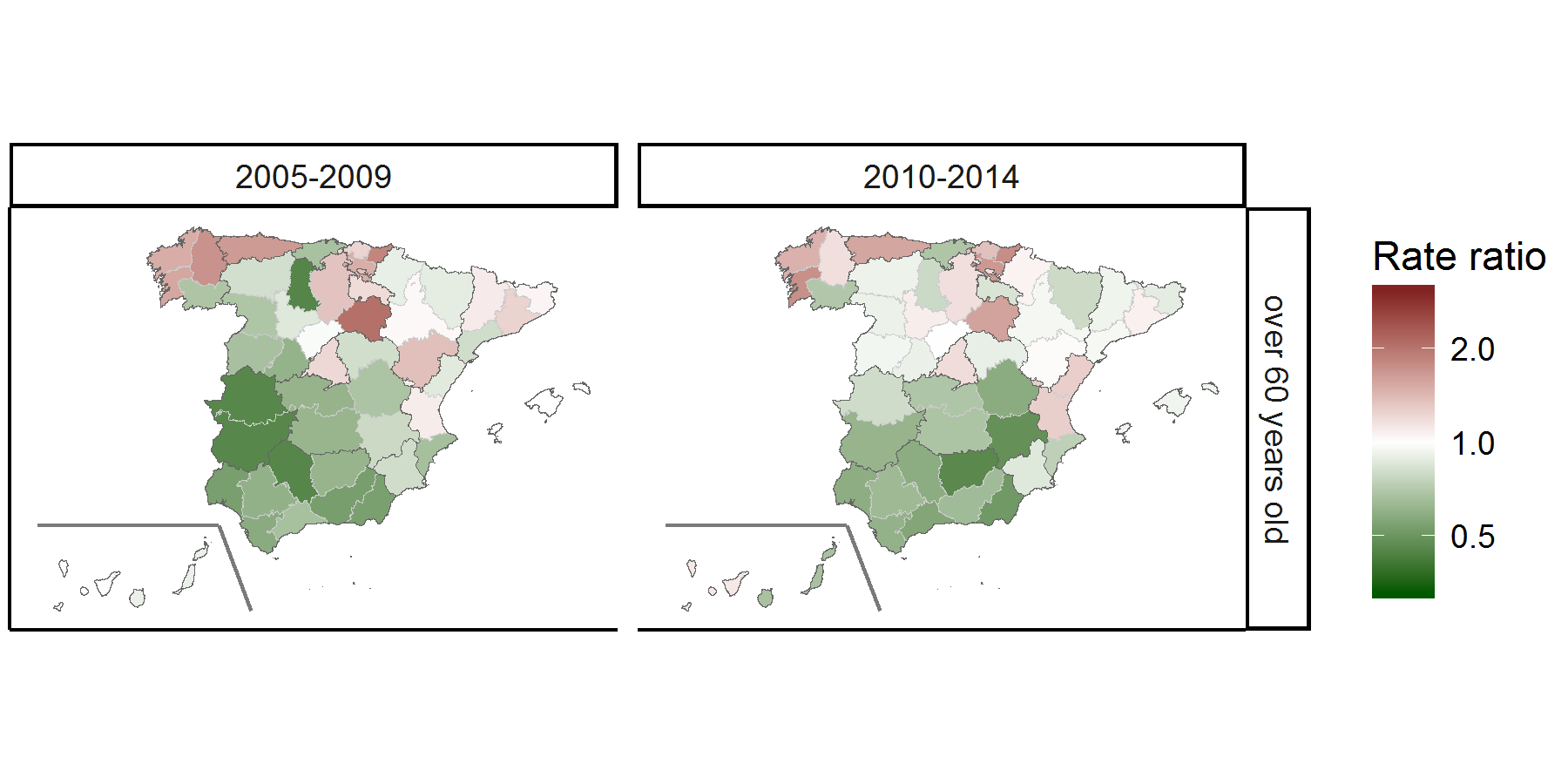

Supplement: S2 Fig — Provincial rate ratio by 5-year period between 2005 & 2014. (TIFF) [file pone.0210980.s002.tiff]
